# New Plants from the Lower Devonian Pingyipu Group, Jiangyou County, Sichuan Province, China

**DOI:** 10.1371/journal.pone.0163549

**Published:** 2016-11-16

**Authors:** Dianne Edwards, Bao-Yin Geng, Cheng-Sen Li

**Affiliations:** 1 State Key Laboratory of Systematic and Evolutionary Botany, Institute of Botany, Chinese Academy of Sciences, Beijing, China; 2 School of Earth and Ocean Sciences, Cardiff University, Cardiff, United Kingdom; Agharkar Research Institute, INDIA

## Abstract

Descriptions of Lower Devonian plants from Yunnan, South China, have revolutionized concepts of diversity and disparity in tracheophytes soon after they became established on land. Sichuan assemblages have received little attention since their discovery almost 25 years ago and require revision. With this objective, fieldwork involving detailed logging and collection of fossils was undertaken in the Longmenshan Mountain Region, Jiangyou County and yielded the two new taxa described here. They are preserved as coalified compressions and impressions that allowed morphological but not anatomical analyses. *Yanmenia (Zosterophyllum) longa* comb nov is based on numerous rarely branching shoots with enations resembling lycophyte microphylls, without evidence for vasculature. The presence of sporangia is equivocal making assignation to the Lycopsida conjectural. The plant was recently described as a zosterophyll, but lacks strobili. These are present in the second plant and comprise bivalved sporangia. The strobili terminate aerial stems which arise from a basal axial complex displaying diversity in branching including H- and K- forms. These features characterise the Zosterophyllopsida, although the plant differs from *Zosterophyllum* in valve shape. Comparisons indicate greatest similarities to the Lower Devonian *Guangnania cuneata*, from Yunnan, but differences, particularly in the nature of the sporangium border, require the erection of a new species, *G*. *minor*. Superficial examination of specimens already published indicate a high degree of endemism at both species and generic level, while this study shows that *Yanmenia* is confined to Sichuan and *Guangnania* is one of the very few genera shared with Yunnan, where assemblages also show a high proportion of further endemic genera. Such provincialism noted in the Chinese Lower Devonian is explained by the palaeogeographic isolation of the South China plate, but this cannot account for differences/endemism between the Sichuan and Yunnan floras. Such an enigma demands further integrated geological, palaeobotanical and palynological studies.

## Introduction

Previous studies on the diversification of terrestrial plants in Early Devonian times in China have largely concentrated on the Lower Devonian assemblages of east and south-east Yunnan Province, [1–4]. These indicate exceptional disparity and a high percentage of endemics, many of which defy attempts to elucidate their affinities. The latter include *Hsüa robusta* [5], *Stachyophyton yunnanense* [6], *Catenalis digitalis*, *Eophyllophyton bellum* [7,8], *Adoketophyton subverticillatum* [9], *Celatheca beckii* [10], *Polythecophyton demissum* [11] and *Bracteophyton variatum* [12]. Scrutiny of species lists in Hao and Xue 2013 [4] shows that, of taxa that could be identified with some confidence, only eight genera out of 28 were cosmopolitan (i.e. were found outside the South China Plate) and that four of these were represented by endemic species (Table 1, this work). Such data hint at the provincialism, as defined by high percentages of endemic taxa, which culminated in the emergence of the late Palaeozoic Cathaysian flora [13], although Xiong *et al*. 2013 [14] found that the Pragian interval was the only one in the Chinese Devonian in which numbers of endemic genera exceeded those of cosmopolitan ones and that post Pragian proportions of endemic genera declined gradually.

**Table 1:** Species list from Geng (1992a, b) and more recently collected fossils. Fossils in lower (L), middle (M) and upper (U) parts of the section. C/E: Cosmopolitan/Endemic, S: Sichuan, G:genus, sp:species, • inaccurate identification.

Taxa	part	Affinites	C/E	References
• *Eogaspesiea gracilis*	L	Unknown	?	Geng 1992a
• *Hicklingia* cf *edwardii*	L	ibid	EG	Ibid
*Oricilla unilateralis* sp. nov	L	ibid	CG, EspS	Ibid
• *Psilophyton* sp.	L	Trimerophytopsida?	?	Ibid
• *Uskiella* sp.	L	Unknown	EG	Ibid
• *Zosterophyllum myretonianum*	L	Zosterophyllopsida	?	Ibid
• *Z*. *sichuanensis* sp. nov.	L	ibid	EG	Ibid
*Z*. *yunnanicum*	L	ibid	EspChina	Ibid
*Amplectosporangium jiangyouense*	L	Unknown	EG	Geng 1992b
• *Drepanophycus spinaeformis*	M	Lycopsida	C	Geng 1992a
• *D*. *spinosus*	M	ibid	C	Ibid
*Drepanophycus* sp	M	ibid	C	Ibid
• *Leclerqia complexa*	U	ibid	?	ibid, Xu et Wang 2007
*Guangnangia* new sp.	U	Zosterophyllopsida	EspS	This paper
*Yanmenia longa* gen. et comb. Nov.	L	Lycopsida?	EG	This paper
*Sciadocillus cuneiformis*	L	Unknown	EG	Geng 1992a

By contrast, the Lower Devonian assemblage of Sichuan province, also on the South China plate/palaeocontinent [15], has been little studied since plants were first reported by Li and Cai in 1978 [16] (*Sporogonites xichuanensis*, *Psilophytites*) and followed by the descriptions of Geng ([17,18]; Table 1). Exceptions are recent papers by Wang in 2007 [19], who described *Zosterophyllum longa* (sic) sp. nov., and Xu and Wang in 2009 [20] who queried Geng’s identification of *Leclercqia complexa*, because the leaves were more frequently branched than in the north American taxon. They did not provide a new name. In view of the recent resurgence of interest in Chinese plants and the availability of new material, a reinvestigation of the Sichuan plants seems long overdue and begins here with the description of two new taxa, including that originally named *Zosterophyllum longa* by Wang in 2007 [19]. A full species list is given in Table 1, where the legend indicates uncertainties of identification and affinity plus taxa in need of revision. Accuracy of identification, plus confidence in dating, is an essential prerequisite to any inferences on the phytogeographic or evolutionary significance of the assemblage.

## Stratigraphy and Age

Deliberations on the age of the plant assemblages in the Pingyipo Group have been hampered by complexity in the geology of the area leading to misunderstandings on stratigraphy, names of strata and correlation. The plant-containing rocks form part of the Tangwangzhai Syncline in the Longmenshan Mountain region where Devonian sequences are found in the east and west limbs in Jiangyou and Beichuan Counties respectively.

The most extensive and relatively well-dated exposures are in a stream section between Guixi and Shawozi villages (Guixi-Shawozi Section, Beichuan County) in the west limb with a possibly complete Devonian sequence, 13 km long in a total thickness of 4700m [21]) of which 2064 m comprises the Pingyipu Group. Although fossiliferous, the fishes, ostracodes, bivalves and brachiopods are endemics but based on conodonts, the strata are considered pre-Emsian. Palynological studies indicate that the the Bailiuping Formation above the Pingyipu group is Pragian, while the group itself ranges from probably the Pragian (Guangshanpo Formation) to probably Lochkovian (remaining three formations). Details of the stratigraphy and fossils are given in Table 2.

**Table 2:** West limb Guixi-shawozi section.

	Formation		Conodont zone	Invertebrates	Fishes
Upper Devonian	Maoba				
	Shawozi				
	Tuqiaozi				
Middle Devonian	Guanwushan				
	Jinbaoshi				
Lower Devonian	Yangmaba				
	Ertaizi		*Polygnathus serotinus*		
	Xiejiawan				
	Ganxi		*P*.*perbonus*,*P*. *dehiscens*		
	Bailiuping				
	Pingyipu Group	Guangshanpo 243m		(Br) *Orientospirifer* cf. *wangi*	*Yunnanolepsis* cf. *chii*, *Tsuifungshanolepsis* cf.*diandongensis*, *Yunlongolepis liui*, *Chuanbeiolepis jiangyouensis*, *Parapetalichthys minoy*
		Guanyinmiao 270m			
		Muerchang 883m		(Br)*Strophochonetes pingyipuensis*, *S*.*convexa*, *Howellella* sp. (Ost)*Guangxinia beichwanensis*, *Beyridia guixiensis*, *Birdscellella sichuanensis*	
		Guixi 688m		(Br)*Lingula longmenshanensis*	
Silurian		phyllitic slate			

**Fig 1:**
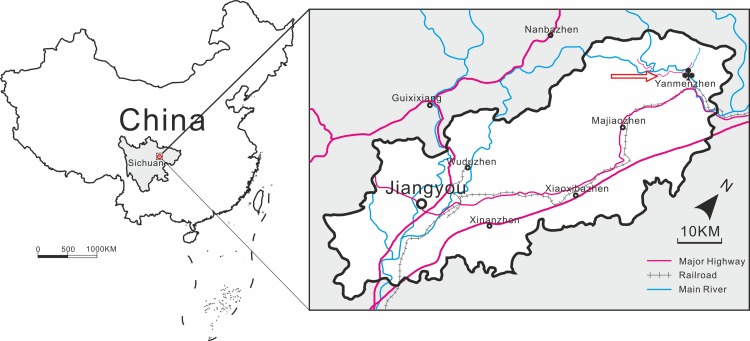
The locality of Pinyipu Group in the Longmenshan Mountain Region showing the fossil site.

Strata near the base of the succession in the east limb, also called the Pingyipu Group and also overlain by the Bailiuping Formation, are exposed in the Yanmenba Section, Jiangyou County about 60 km from the west limb (Fig 1). The underlying rocks are middle Silurian. However the lithology of the Pingyipu Group and succeeding strata differ in the two limbs such that the formations named in the west cannot be identified in the east. This makes correlation, and hence age determinations of the plant fossils, equivocal.

The Pingyipu Group in the Yanmenba section is about 672 m thick, considerably less than the same group (2084 m) in the Guixi-Shawozi section. Two sedimentological logs have been produced. That concentrating on fish and spore localities was published in Pan and Wang in 1978 [22]. The one below was measured by Geng and Li during the collection of the new fossil plants.

**Table:** 

The sequence of rocks is described as follows.	
Pingyipu Group (Pragian to Lochkovian )	691.7m
Upper part:	
13. dark grey siltstone, with fossil plants (**Horizon 3**)	1.5m
	
Middle part	
12. yellow and white sandstone with muddy siltstone at upper part	186.4m
11. dark grey siltstone	41.1m
10. greyish white quartz sandstone	96.7m
9. dark grey siltstone	31.4m
	
Lower part	
8. yellow and greyish white quartz sandstone	49.9m
7. dark grey quartz sandstone	39.4m
6. grey quartz sandstone inter-bedded with variegated muddy siltstone	21.7m
5. yellow and greyish white quartz sandstone inter-bedded with dark grey siltstone	76.3m
4. grey quartz sandstone with yellow sandstone at upper part	50.9m
3. light brown and greyish white quartz sandstone inter-bedded with yellow silty mudstone, with fossil plants (**Horizon 2**)	42.2m
2. greyish white quartz sandstone inter-bedded with grey muddy siltstone	45.5m
1. light brown quartz sandstone inter-bedded with dark grey muddy siltstone, with fossil plants (**Horizon 1**)	8.7m

Pan and Wang divided the section into three parts. The lowest part (about 300 m thick; their beds 1–3, here beds 1–8) contains the petalichthyid fish, *Neopetalichthys yenmenpaensis* Liu. The middle part (c.360 m; their 9–17, here 9–12) has yielded galeaspids: *Dongfangaspis major*, *Lungmenshanaspis kiangyouensis* Pan et Wang, *Sinoszechuanaspis yanmenpaensis* Pan et Wang, *Sanqiaspis rostrata* Liu, *S*. *sichuanensis* Pan et Wang, and the petalichthyid, *Xinanpetalichthys shendaowanensis* Pan et Wang [22,23]. The fishes, *Donfangaspis* and *Sanqiaspis rostrata* were thought to indicate a Pragian age. Hence the lower part of the group was considered to be Siegenian/Pragian and possibly partly Gedinnian/Lochkovian in age ([23,24,25,26,27] and also in Fig 1 of Turner et al., 1995 [28]). However all the fish species are endemic making global correlation less secure. Abundant spores found in the middle part of the Yanmenba section(Pan and Wang’s bed 12 probably equivalent to bed 11 here) include *Punctatisporites*, *Retusotriletes*, *Granulatisporites*, *Apiculiretusispora*, *Acanthotriletes*, *Lophotriletes*, *Lophozonotriletes*, *Emphanisporites*, *Convolutispora*, *Stenozonotriletes*, *Archaeozonotriletes*, *Laevigatosporites*. The absence of species names hampers age determinations [22], especially as better known Pragian species show little disparity based on subtle differences. A similar assemblage has been found in the lower part of the Nagaoling Fm., Guanxi, South China and regarded as Lower Pragian [23]. However, in describing a spore assemblage from the Xujiachong Formation from Qujing as similar in composition to those in the early Pragian-early Emsian *polygonalis-emsiensis* Spore Biozone of Laurussia, Wellman et al. in 2012 [29] emphasised the problems of correlation over long distances based on spores especially considering the isolated position of the South China plate, the high proportion of endemics occurring there and the lack of biostratigraphically useful, independently co-occurring fossils. We endorse these reservations in attempts to date the Sichuan fossils.

In conclusion, based on stratigraphy and palaeontology, we conclude that the Pingyipu Group in the Yanmenba section is best considered Lochkovian to Pragian in age.

Fossil plants were originally discovered by Geng at three horizons (Lower, Middle and Upper in Table 1; beds 1, 3 and 13 here) in the outcrop on the road running parallel to the dammed Yanmen river, near Yanmenba village. One of the taxa described here comes from bed 1 and hence might be Lochkovian and the other (*Guangnania*) is much younger (13) and most likely Pragian

## Material and Methods

The plants are preserved as easily dislodged compressions comprising granular to glossy sheets of coalified material. The latter specimens may be completely flat but others show some topographic features. Some fossils display an orange halo. The matrix a grey, fine-grained sandstone, is exceedingly hard, and splits with uneven fracture and no obvious bedding planes. The matrix with *Guagnania* is much harder and possibly with larger grain size than the one with *Yanmenia*. Individual plants may extend through several millimetres of rock, such that with the exception of some leafy stems, splitting rarely revealed part and counterparts. Most of the fossils are fragmentary sterile axes which, except for those with diagnostic spines or enations, are impossible to identify. They rarely show a preferred orientation. An exception is the almost complete new zosterophyll with fertile and almost complete basal rhizomatous system still present. This suggests that the plant had not been transported a great distance from where it grew. There are no marine animal micro- or macrofossils at the locality, and sedimentary structures that might indicate fresh water deposition are lacking. A lacustrine rather than fluviatile depositional environment is thus perhaps a more likely possibility.

Conventional palaeobotanical techniques (eg. maceration and oxidation in Schulze’s solution) failed to yield residues showing anatomical detail. Further morphological detail was obtained by removing grains of the matrix using tungsten needles sharpened by dipping into molten sodium nitrite, but this was hampered by the hardness of the matrix. Photography utilised polarised light. Scanning electron microscopy of whole uncoated pieces of rock was facilitated by a FEI XL30 ESEM FEG microscope (Philips, Eindhoven, The Netherlands) with environmental chamber. No anatomical detail was seen on the surfaces of coalified material, but traces of outlines of epidermal cells were noted on the matrix where coalified material was missing.

All specimens are deposited in the Institute of Botany, Chinese Academy of Sciences, Beijing.

## Nomenclature Acts

The electronic version of this article in Portable Document Format (PDF) in a work with an ISSN or ISBN will represent a published work according to the International Code of Nomenclature for algae, fungi, and plants, and hence the new names contained in the electronic publication of a PLOS ONE article are effectively published under that Code from the electronic edition alone, so there is no longer any need to provide printed copies.

The online version of this work is archived and available from the following digital repositories: PubMed Central, LOCKSS, Institute of Botany, Chinese Academy of Sciences.

## Palaeobotanical Analyses

### *Yanmenia* (*Zosterophylum*) *longa* comb. nov (Figs 2A–2S and 3A–3J)

**Fig 2:**
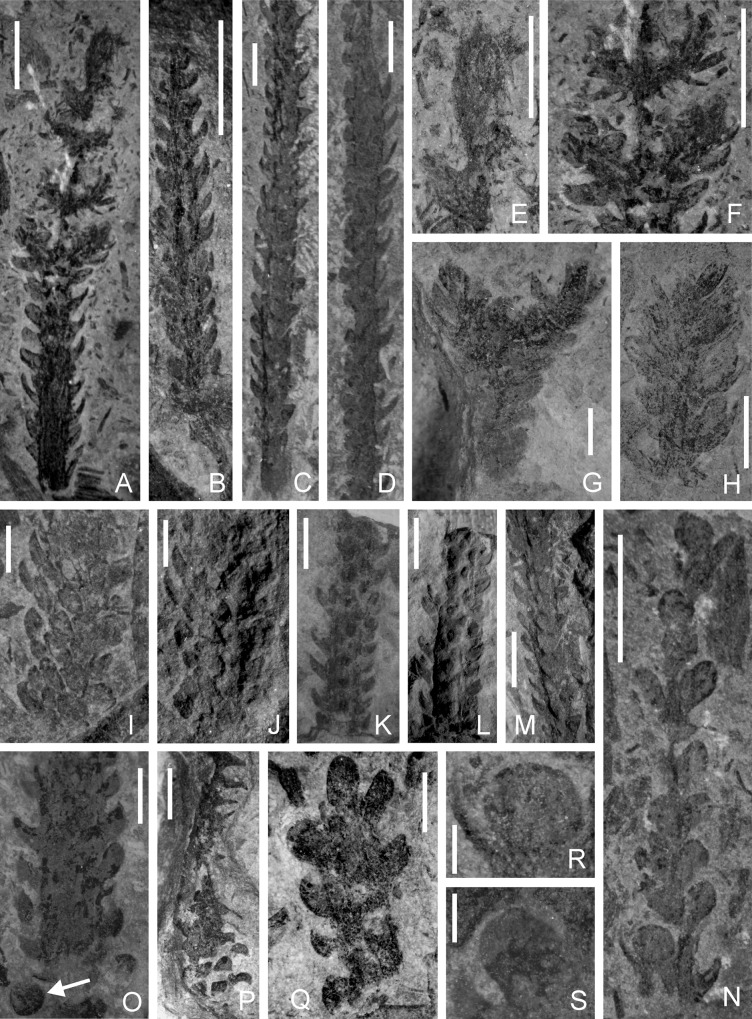
A-S *Yanmenia* (*Zosterophyllum*) *longa* gen. et comb. nov., Yanmenba, Jiangyou, Sichuan, SW China. A. Leafy specimen with intact tip and lateral branches. Specimen Number: 9255b. Scale bar = 5 mm. B. Leafy specimen showing decrease in size of leaves and overall diameter of shoot distally. Specimen Number: 9252a. Scale bar = 5 mm. C, D. Parallel-sided shoots, incomplete at both ends. Note exposed stem in proximal region in C. Specimen Number: 9261, 9231. Scale bars = 4.5 mm, 4mm. E. Recurved shoot apex magnified from A. Scale bar = 5 mm. F. Opposite pairs of shoots, leaf arrangement crowded and indistinct, magnified from A. Scale bar = 5mm. G. Bifurcating axis with crowded indistinct leaves. Specimen Number: 9243. Scale bar = 2mm. H. Apex with crowded indistinct leaves. Specimen Number: 9257. Scale bar = 5 mm. I,J. Leafy specimen with surface exposed, photographed in even (I) and unilateral (J) illumination. Latter shows relief of at least four rows of leaves. Specimen Number: 9260. Scale bars = 3 mm. K,L. As for I, J, but leaves represented on surface by vertically elongate mounds. Specimen Number: 9247. Scale bars = 5mm. M. Leafy shoot in unilateral illumination, some leaves appearing as longitudinal ridges. Specimen Number: 9267. Scale bar = 4.5 mm. N. An atypical leafy shoot with widely spaced lateral appendages, some of which may be sporangia (See R,S). Specimen Number: 9228. Scale bar = 5 mm. O. Wide stem, with differently compressed leaves attached at margins. Arrow indicates detached possible leaf or sporangium. Specimen Number: 9224.Scale bar = 2 mm. P. Twisted shoot. Note tips of leaves exposed proximally. Specimen Number: 9252. Scale bar = 5 mm. Q. Fragment of shoot with distal leaf preserved in face view with wide attachment. Specimen Number: 9268. Scale bar = 3 mm. R,S. Possible sporangia based on relatively large width/height ratio and possible axillary relationship with leaf. Magnified from N. Scale bar = 1 mm.

This description is based on about 30 specimens of leafy shoots whose gross morphology in the majority of cases appears remarkably uniform. Shoots are usually unbranched with very straight parallel sides, and fall into a narrow range in diameter (Fig 2A–2D). They give an impression of rigidity and thus suggest they derive from a plant with an erect growth habit. We use the term *leaves* here to describe the bilaterally symmetrical, flattened, lateral organs and will discuss their botanical nature below. The longest unbranched specimen reached c. 50mm, and is incomplete at both ends. Branching has been observed in only two specimens. One (Fig 2G) shows a terminal± equal bifurcation where appendages are less well ordered and individual leaves are less distinct. In the second (Fig 2A, 2E and 2F), which is 35 mm long, three opposite pairs of branches inserted perpendicular to the stem occur below an intact apex (Fig 2A and 2F). Again leaves are crowded and less regular. The stem apex is slightly recurved (Fig 2E). Similarly sized fragments lacking evidence of regular leaf insertion are found isolated in the rock matrix (Fig 2H) and may represent a form of vegetative reproduction. Alternatively they may have been less robust than the rest of the shoot and broken away during transport.

**Fig 3:**
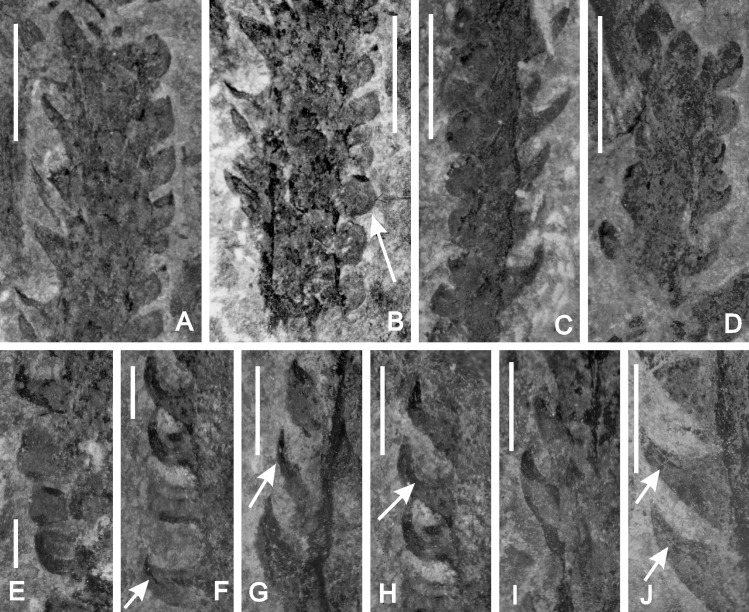
A-J. *Yanmenia* (*Zosterophyllum*) *longa* gen. et comb. nov. Yanmenba, Jiangyou, Sichuan, SW China. Leafy shoots showing variation in appearance of leaves depending on orientation and compression. Scale bars in A-D = 5 mm; in E-J = 2mm. A,B. Leaves on left in profile probably show lateral compression, those on right (arrow), show minimal folding. Specimen Number: 9250. C. Specimen Number: 9260. D. Distal leaves in face view. Specimen Number: 9259. E-J. Arrows (F,H,J) indicate margin of partially folded leaf and (G) a laterally compressed leaf. Specimen Number: E,F- 9287; G—9292, H- 9287, I- 9232

Stem diameter (excluding leaves) ranges between 2.4 and 3.5mm, with little change along the length of a specimen. An exception (Fig 2B) shows slight tapering distally (to c.1.6 mm) but the tip is incomplete. Details of the stem surface are usually completely obscured by the leaves, although unilateral illumination occasionally shows surficial longitudinal ribbing (Fig 2A Basal regions of the longer specimens sometimes lack the regular arrangement of leaves (Fig 2C).

The leaves themselves are best seen on the sides of the stems, where they display a variety of shapes produced during their compression (Figs 2 and 3). Greater uniformity is seen in a single vertical row.

Specimens illustrated in Fig 3A–3C show such differences with outlines ranging from falcate to roughly triangular to oval, but all with a broad base at attachment to the stem. Length of attachment is between one and two mm (x = 1.4, n = 42). A leaf stalk is never present. Such bases are ± contiguous and produce oval to sub rhomboidal mounds on the stem surface, best seen in unilateral illumination (Fig 2I–2M).The falcate shapes (e.g.Fig 3F–3H) were produced when leaves attached to the side of the stem perpendicular to the plane of compression were tangentially compressed (i.e. not folded) so that the lamina is obliterated. The shape thus approximates to the outline of a longitudinal section through the centre of the leaf. Such compression nicely demonstrates the decurrent base and the decrease in thickness of the lamina distally. The remaining outlines in profile result from the extent of folding of the lamina during compression (cf. Fig 3E–3J). In a few cases, a line marks the margin of the smaller part (Fig 3F, 3H and 3J). Further variation is seen in the angle of the lamina to the stem. In most cases the lamina is curved distally, such that it will overlap the base of the leaf above, and becomes almost parallel with the stem. In some, the lamina is almost perpendicular to the stem and recurved distally (Fig 3F). The shape of the lamina in face view is more contentious as preservation is rarely seen in this plane. Examples are illustrated in Fig 2Q and 2N; 3D with shapes ranging from almost hemispherical to elliptical to slightly acuminate, the first (9268) possibly relating to a more distal position on the stem apex. Leaf apices are rounded (Fig 2P and 2Q).

Because of slight overlap and rare occurrences of face views, lamina maximum width is based on relatively few examples (Fig 2N, 2O and 2Q). Lamina length, as measured between its tip and the base of the attachment site is more reliable (range 0.8 to 3.2mm; x = 2.2, n = 39). Neither midrib vein nor vascular trace has been observed. Leaves are inserted in a very low angled helix (almost whorled) with usually six leaves per gyre. This is based on the number of bases (mounds) on the stem surface and those at the edge. Rare examples possess at least eight leaves/gyre (Fig 2I).

Many of the specimens show more or less elliptical structures arranged in rows but based on their relationships with rows of more typically folded leaves, we interpreted them as leaves and not sporangia (Fig 3B–3E). A single example (Fig 2N) shows more staggered departures, less regularity in leaf arrangement and more constricted leaf bases. These may be sporangia, although there is no change in their thickness of coal which would reflect the increased volume of a sporangium nor any evidence of marginal features. The best preserved is associated with a typical falcate leaf (Fig 2R), but from the orientation and quality of preservation, it cannot be confirmed as axillary. The elliptical structure is 1.6 mm high and 1.9 mm wide, and thus differs from the leaves where, in face view, height is greater than width (x = 1.9 mm high; x = 1.2 mm). A second uncovered example (Fig 2S) occurs with more typically shaped leaves. The isolated elliptical structure seen below a leafy stem arrowed in Fig 2O may be an isolated sporangium. Such uncertainties in confirming the presence of unequivocal sporangia caution against inclusion of fertile characters in the diagnosis.

## Systematic Palaeobotany

Plantae Incertae sedis

*Genus*: *Yanmenia* gen. nov.

[urn:lsid:ipni.org:names:XXXXX]

*Type species*: *Yanmenia longa* (Wang) Edwards *et al*. comb.nov.

[urn:lsid:ipni.org:names:XXXXX]

*Diagnosis*: Herbaceous leafy stems with distal branching isotomous or lateral in opposite pairs. Simple entire persistent leaves with decurrent broad bases and bifacial laminae, almost circular to elliptical in face view. Laminae directed distally. Leaves attached to all sides of stem in a low helix, 6 or rarely 8 per gyre.

Derivation of name: From the river close to the locality.

*Type species*: *Y*. *longa*

*Basionym*: *Zosterophyllum longa* Wang 2007. *Acta Geologica Sinica*, 81: 525–538, Plate 1,3–11; Fig 1ª

*Holotype*: CS1

*Diagnosis*: As for genus. Plant at least 50mm high. Relatively unbranched? rigid stems 2.4–3.5mm wide. Rare branching isotomous or as opposite pairs. Decurrent leaf attachment sites 1.0–2.0mm high, total leaf height 0.8–3.2mm. Lamina width c.1.2mm. Leaf bases contiguous. 6–8 leaves per gyre.

*Locality of holotype*: Yanmenba section, 32° 13.87’N, 105° 10.98’E, Jiangyou County, Northern Sichuan.

### Synonymy with *Zosterophyllum longa* D Wang 2007 [19]

In 2007 Wang [19] described identical specimens from the Yanmenba Section, (citing Geng’s locality data [17]) as the strobili of a new species of *Zosterophyllum*, although his specimens were collected at another nearby locality. He interpreted the enations, here described as leaves, as sporangia and some branching smooth axes, found isolated in the same matrix, as subtending stems. However in common with our observations he found little direct evidence for sporangia (‘Sporangial thickenings and stalks hardly visible’ p.527). Dimensions in his diagnosis and very brief description fall within those we have obtained and details of his ‘sporangia’ with those of our leaves, although his specimens are more informative of the structures in face view. Wang designated CS1, Plate 1–6 as the holotype, now placed in the new combination, *Yanmenia longa*. It is housed in the Department of Geology, Peking University, Beijing.

## Affinities

The Sichuan material comprising sparingly branched shoots bearing simple, regularly inserted, isomorphic enations lacking vasculature falls into a small group of Silurian/Devonian plants which, in their vegetative state, are impossible to classify unequivocally. Examples include *Bowerophylloides mendozaensis* from the Lochkovian of Argentina [30], originally named *Baragwanathia* [31], Přídolí *Lycopodolica* and *Protosawdonia* from Podolia [32], a single ‘leafy axis’ from the Pridoli of Xingjiang [33] and Pragian *Hueberia zhichangensis* from the Posongchong Formation [34]. All of these lack broad laminae. In some cases, even an animal or algal affinity is a possibility (see discussion in Edwards et al. 2001 [30]).

Particularly frustrating has been our inability to demonstrate the presence of a microphyll in *Yanmenia* or the nature of associated structures as sporangia. The possibility remains that a vascular strand did not persist even in such apparently well-preserved material or, as was the case for *Asteroxylon*, it did not extend into the leaf lamina. However, even if a lycophyte, in its simple, entire, bifacial leaves it differs from those of coeval examples (See Hueber 1992, Figure 11 [35]). Leafy liverworts and mosses have bifacial leaves, but these do not possess swollen, presumed multicellular bases, while the size of the subtending stems proportional to the leaves is far greater than in extant examples of mosses. On balance we are persuaded towards lycophyte affinity. A further similarity, if we are correct in our interpretation, are the similarities of the small lateral branches called bulbils in *Huperzia selago*.

### *Guangnania minor* sp. nov. (Figs 4A–4D and 5A–5I)

**Fig 4:**
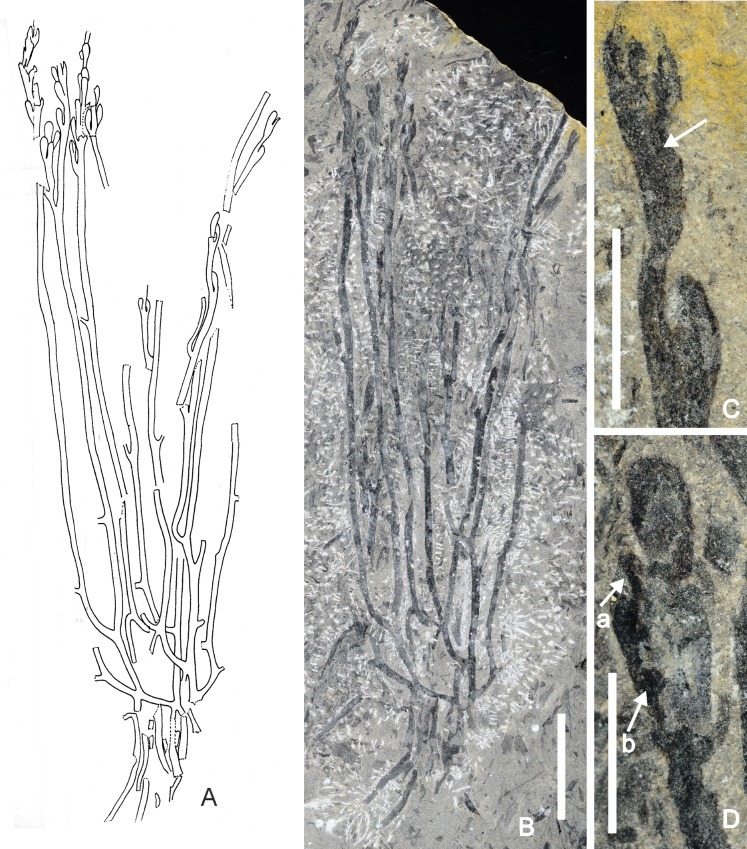
A-D *Guangnania minor* sp. nov. Yanmenba, Jiangyou, Sichuan, SW China. A. Line drawing of holotype Specimen Number: 9402. B. Entire specimen (holotype) after development (uncovering). Arrow indicates isotomous branching. Scale bar = 10mm. C,D. Parts of strobili magnified from B. C. Arrow indicates ridge beneath sporangium, representing stalk adpressed to strobilar axis. Note narrow diameter of strobilar axis between sporangia. Scale bar = 5mm. D. Arrow a marks ridge, arrow b, a decurrent stalk. Upper left hand sporangium has concave surface and lies below level of strobilar axis. Scale bar = 4 mm

**Fig 5:**
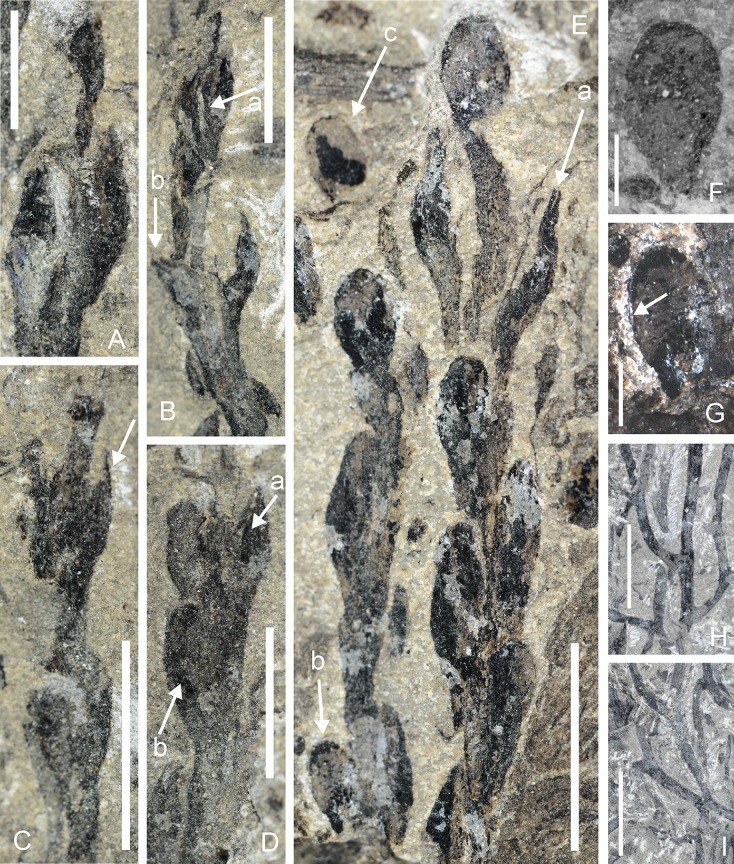
A-I. *Guangnania minor* sp. nov. Yanmenba, Jiangyou, Sichuan, SW China. A-D. Parts of strobili magnified from holotype (Fig 4B). A. Sporangia compressed laterally: left hand example has convex surface. Scale bar = 3.mm. B. Arrow a indicates ridge, arrow b, separating valves. Scale bar = 5 mm. C. Note narrow strobilar axis distally; arrow indicates fractured valve. Scale bar = 5 mm. D. Arrow a marks ridge, arrow b, decurrent stalk. Upper left hand sporangium has concave surface and lies below level of stobilar axis. Scale bar = 4 mm. E. Specimen shows two aligned partial strobili, with sporangia in dorsiventral and lateral views. Arrow an indicates distal separation of the valves, arrow b, narrow marginal features. Specimen Number: 9291a. Scale bar = 5 mm. F. Isolated sporangium in dorsiventral view. Counterpart indicated by an arrow (a) in E. Specimen Number: 9291b. Scale bar = 1 mm. G. Sporangium from base of strobilus (arrow b in E) with faint dark line at margin (arrow). Scale bar = 1 mm. H,I. Enlarged base of holotype showing range in branching. Scale bars = 10mm.

This description is based on two specimens. The larger is a spectacular fossil at least 155 mm long with both distal and basal branching systems preserved (Fig 4A and 4B). They are separated by naked parallel-sided axes of a diameter that is remarkably uniform along their length (1.0–1.6 mm wide, n = 21, x = 1.3; majority are 1.2 and 1.4 m) with infrequent and isotomous branching with some overtopping. Bifurcation may occur close to the fertile region arrowed in Fig 4B). The latter is more common in the basal regions, where it is accompanied by K configurations and occasional, apparently short, unbranched laterals with rounded apices (Fig 5J and 5I), although the majority are probably broken ends. Dimensions are similar to those in the axes preserved in parallel. These are assumed to have been erect. Although the branching complex is preserved in more or less the same plane as the axes (stems) bearing strobili, it is assumed that the majority formed a horizontal rhizomatous complex, similar to that seen in *Zosterophyllum* [36–39]. These fertile regions comprise strobili preserved more or less in parallel. The longest strobilus attains 30 mm and is incomplete distally. Sporangia are lateral and arranged helically, although their exact disposition is unknown. Tips of sporangia are at approximately the same level (e.g. Fig 4C), but sporangial insertion shows very small vertical spacing (up to three consecutive stalks observed) such that they are not opposite or verticilate. Slightly vertically extended mounds or depressions probably represent superficial insertion of sporangia which are not preserved. Strobili on the larger specimen show considerable relief, extending through some depth of sediment such that sporangia are fractured in different planes. Thus the area arrowed in Fig 5C, results from fracture rather than actual tapering of the sporangium. The sporangia themselves are variously compressed. In the more complete specimen, the majority is attached to the sides of the strobilar axis—rare examples are partially obscured by the latter and when this is removed central areas of the sporangium are concave as if adaxially curved around it. Those preserved in presumed lateral view show no folding but sometimes bear a vertical ridge or depression over which the coalified material is continuous (arrows in Figs 4D and 5D). By contrast, in the second, smaller specimen (Fig 5E) a number of sporangia are preserved dorsiventrally, either in full face view or with limited asymmetry. Such sporangia are vertically elliptical (1.3–1.9 mm wide; x = 1.7) and taper without interruption into a stout stalk. The majority of sporangia in both specimens are preserved in profile and again show no distinction from the stalk (Figs 4C, 4D and 5A–5D). There is no evidence of pronounced curvature: the stalks are inserted at an acute angle, are decurrent and, where superimposed on the strobilar axis, appear as broad ridges or depressions (Fig 4C). Stalk width is c.0.7 mm (0.6–0.9 mm) and up to 3mm where free, but the latter is an approximation. The combined height of stalk and sporangium is 2.8–3.5mm (x = 4.1, n = 9). Sporangial height is about 2.5 mm. There is little change in sporangial dimensions between base and distal regions of the strobilus. Evidence for two valves comes from sporangia in profile that show separation of coalified layers at c.0.4 mm from their tips (arrows in Fig 5B and 5E) and is interpreted as evidence for partial dehiscence. The abaxial part is continuous with most of the rest of the sporangium which is interpreted as the abaxial valve. Some specimens preserved dorsiventrally show a very narrow line around the distal margin, but this is not comparable to the marginal fractures noted in zosterophylls [40] nor does it equate to the marginal split recorded above. Further evidence for two valves may derive from the change in topography of the sporangium about a third of the diameter from the adaxial limit, perhaps indicating a bivalved sporangium compressed laterally with smaller adaxial valve and possibly a more resilient marginal feature, but there is no evidence of any variations in the thickness of coalified material in this region. Sporangia preserved in face view show no marked asymmetry in size of valves. Separation of two coalified layers during uncovering of the presumed sporogenous area provides further evidence of two superimposed valves.

## Systematic Palaeobotany

Subdivision: Lycophytina *sensu* Kenrick and Crane 1997 [41]

Class: Zosterophyllopsida Hao and Xue 2013 [4]

Order: Incertae sedis

Genus: *Guangnania* Wang and Hao 2002 [42]

Type species: *Guangnania cuneata* Wang and Hao 2002 [42]

*Guangnania minor* sp nov Edwards, Geng and Li

[urn:lsid:ipni.org:names:XXXXX]

Holotype sic designates specimen CBYn9402, IBCAS, Beijing Fig 4B; (Holotype; F[F-XXXXX], US [US-XXXXX])

*Diagnosis*: As for genus, but see discussion below. Plant at least 155 mm tall with dichotomously branched, smooth, erect axes, 1.2-(1.3)-1.4 mm, wide and basal axes 1.2-(1.35)-1.6 mm wide with K- and dichotomous branching. Strobili at least 30 mm long and incomplete distally, comprising helically arranged, irregularly spaced sporangia; strobilar axes 0.6-(1.2)-1.6 mm wide. Sporangial stalks inserted at an acute angle, decurrent and partially fused to the strobilar axis, free parts almost straight; length of latter 0.7–1.3 mm and width, 0.6-(0.72)-0.9mm. Combined stalk and sporangial length (junction usually not well-marked) 2.9–5.5 mm. Erect sporangia, with small adaxial curvature, cuneate in face view, tapering into broad stalk. Marginal thickening very narrow or indistinct. Valve in face view 1.2-(1.7)-1.9 mm; height approx. 1.7–3.2 mm.

*Locality*: Yanmenba section, Longmenshan Mountain Region, Jiangyou County, Northern Sichuan.

*Stratigraphy and Age*: Pingyipu group; Lochkovian–Pragian.

*Notes*. The fossil described above comprises both fertile and basal regions and thus joins the small group of Devonian whole plant compressions that includes of compression fossils includes Middle Devonian *Hicklingia edwardii* from Scotland [43], Přídolí *Cooksonia* from the Barrande and the most complete of all, *Zosterophyllum shengfengense* from the early Devonian Xitun Formation, Qujing Province, Yunnan [39]. The aerial parts of the latter plant have a tufted appearance produced by numerous stems, terminating in strobili, that radiate from a downwardly directed tuft of fibrous root-like axes.

## Identification and Affinities

In their lateral sporangia inserted on short stalks, their terminal strobili and the complexly branching basal region, the Sichuan plants share characters with the zosterophyll-lycophyte clade, although anatomical detail and unequivocal evidence of its bivalved sporangia are needed to confirm this. The vertically extended sporangial shape precludes its assignation to *Zosterophyllum*, a genus with numerous representatives in the Lower Devonian of Yunnan Province [39,40,44] and elsewhere in South China, or to other members of the zosterophyll clade. However *Guangnania cuneata*, a Lower Devonian species endemic to south-east (Pragian Posongchong Formation) and east (Pragian–Early Emsian Formation) Yunnan [42], has vertically elongate bivalved sporangia arranged in lax spikes and is associated with H-type branching axes. There are differences in the nature of the sporangia as characterised in the generic diagnosis of *Guangnania*. These include the presence of a conspicuously thickened sporangial border, pronounced asymmetry of the valves with marked adaxial curvature, and curved sporangial stalks. In the Sichuan specimens a very narrow border may be present and the adaxial valve is only slightly smaller. Removal of the strobilar axis reveals a gentle concavity in the adaxial valve, while the stalks are almost straight. Some of these differences are essentially qualitative, and also could be related to different modes of preservation. The new specimens are more heavily coalified and homogenised and occur in coarser sediments thus obliterating morphological detail. Particularly relevant here to the generic diagnosis is the presence of the marked border in *G*. *cuneata*. We recently discussed this zosterophyll feature [40] and concluded that there is variation in border thickness in the genus *Zosterophyllum* even in the type species, *Z*.*myretonianum* (where possibly preservational), and this might be the case for *Guangnania*. Thus because the nature of the sporangial border remains unresolved, we have placed the Sichuan plants in the genus, *Guangnania*, but as a new species, based on differences in the relative length to width of the sporangia, differences in relative size of the two valves and their curvature and the compactness of the strobili. The Sichuan dimensions (Table 3) are generally smaller than those originally described for *G*. *cuneata*, but the gap has been lessened by the more recent discovery of a further smaller specimen of the type species from the Zhichang section of the Posongchong Formation [4]. This possesses greater sporangial height compared with width than in the type material. (Dimensions in brackets in Table 3). Wang and Hao were convinced that the sporangia themselves possessed adaxial curvature in life rather than displaying folding during compression. The authors' illustrations indicate marked differences between the specimens collected from the Posongchong Formation, near Daliantong Village (WH-1), Plates I & 11 and the Xujiachong Formation, near Xujiachong village, Plate Ill although dimensions are similar. The Sichuan specimens are superficially closer to the latter.

**Table 3:** Comparison of *Guangnania cuneata* and G. sp nov. All dimensions in mm.

	*Guangnania cuneata*	*Guangnania minor*
Maximum length	80	155
Stem width	0.6-(*2.0)-3.0 (*0.9–1.8)	1.2-(1.3)-1.4 (erect, n = 9)
		1.2-(1.35)-1.6 (basal, n = 7)
Strobilus length	>73	>30
Strobilus axis width		0.6-(1.2)-1.6 (n = 6)
Sporangia face view		
height	3.5-(*6.8)-9.6 (*3.9–6.2)	c. 1.7- c. 3.2
width	1.2-(*2.1)-2.8 (*1.5–1.9)	1.3-(1.7)-1.9 (n = 8)
Sporangial width in side view		0.8-(1.1)-1.6 (n = 6)
Stalks length	1.5-(*3.2)-5.2 (*1.2–1.8)	0.7–1.3 when free
Stalks width	0.3-(*0.6)-1.3 (*0.6)	0.6-(0.7)-0.9 (n = 9)
Angle of attachment	20°-40° (*40°)	Acute

* specimen measurements from Zhichang section.

Finally, comparisons might be appropriate with another putative zosterophyll, *Hicklingia edwardii*, from the Middle Devonian of Scotland [43], already mentioned because of the completeness of its preservation and indeed Geng’s record in the Sichuan assemblage which requires reinvestigation. However Edwards in 1976 [45] had described some new Scottish isolated strobili in which lateral bivalved sporangia were inserted in a loose helix. In face view, the sporangia are almost isodiametric,—the limits of each being defined by two very narrow coalified lines which indicate that the adaxial valve was very slightly smaller than the abaxial one. Sporangia are borne erect on stout straight or slightly curved stalks. Thus apart from the three dimensional construction of the sporangia, and the marked distinction between sporangium and stalk in *Hicklingia*, the Chinese and Scottish plants are architecturally similar.

In their account of *Guangnania*, Hao and Xue in 2013 [4] commented on similarities in sporangial shape with those of another plant in the Posongchong Formation, *Yunia dichotoma* [7] which they identified as a zosterophyll on the discovery of a further species, *Y*. *guangnania*, in the same formation whose sporangia were borne laterally [4]. The very large sporangia (5.0-(6.7)-8.3 mm long; 2.2-(3.0)-4.8 mm wide) were bivalved with evidence of distal dehiscence, but no thickened border, and, although elongate elliptical in face view, they were borne on short stalks and scattered. They are thus quite distinct from the Sichuan examples.

To sum up, the Sichuan specimens are considered closest to *Guangnania cuneata*, but differ from the Yunnan species principally in the absence of a pronounced sporangial border. We therefore place them in a new species *Guangnania minor*.

## Sichuan Palaeogeography

**Fig 6:**
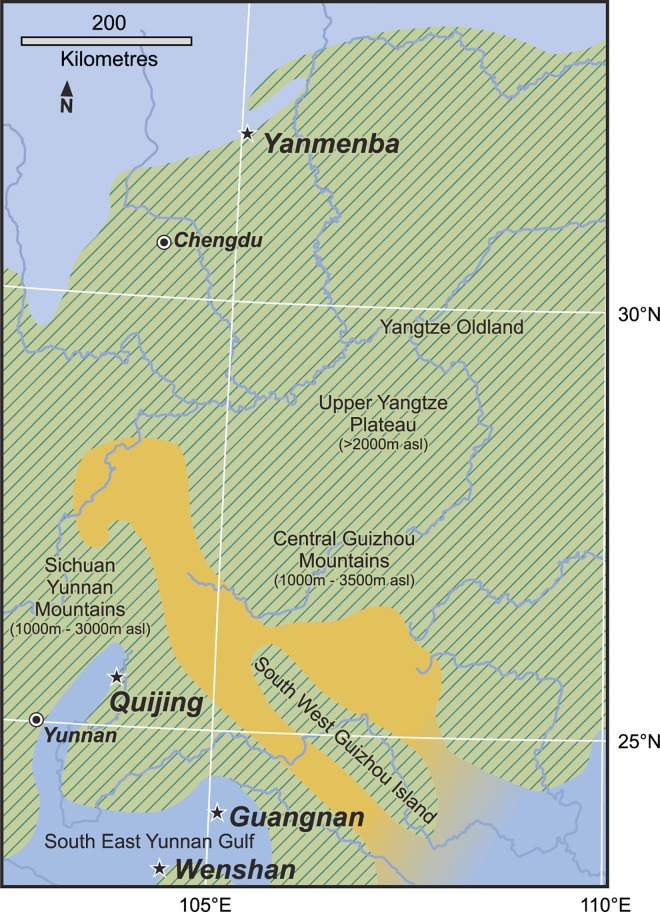
Palaeogeography map of part of South China plate, showing major plant localities. Green represents land, blue, shallow seas and yellow estuarine conditions.

One of the most important developments in Devonian palaeobotany over the past thirty years relates to increased knowledge on Chinese vascular plants and the demonstration of their amazing disparity, recently summarised for the Posongchong flora of Yunnan [4], when compared with especially Pragian plants of Laurussia. Such studies indicate a high proportion of endemics on the southern part of the South China plate, explained in part by its relative palaeogeographic isolation from Laurussia, although at broadly the same latitudes [46]. As we begin to revise the Lower Devonian assemblages from Sichuan, we are again impressed by the high proportion of endemic taxa but note that these are not the same as those in Yunnan (Table 4). Such differences require explanation as the two floral ‘zones’ are roughly coeval. Fig 6 shows that the Sichuan locality is located towards the northern part of the South China plate geographically separated today by some 700 km from the Yunnan ones. A more detailed insight into the Devonian palaeogeography of Yunnan, which is seen on Fig 3.1 in Hao and Xue 2013 [4], towards the southern margin of the plate indicates great complexity in coastline and islands following marine transgression and faulting, that would have produced a wide range in topography and habitats potentially suitable for plant colonisation. It is noteworthy that many bedding planes are covered by a single taxon. By contrast the fossils in Sichuan are often more fragmentary and occur as isolated examples in mixed assemblages. Detailed examination of lithofacies has not yet been undertaken that might indicate completely different source areas from those in the south. However this could only be part of the explanation. We note that there were remnants of ancient mountain chains (Fig 6, [46]) on the intervening land area, but at estimates of heights less than 3000m [15], these would not have been a major barrier to spore dispersal. Changes of temperature with altitude would have been minimal considering the tropical position of the plates although there is the possibility of differences in hydrological regimes.

**Table 4:** Species comparisons between the Posongchong Formation, Yunnan and the Pingyipo Group/Formation, Sichuan.? = uncertain affinity, * = further investigation required, (L) = Lochkovian.

Group	Taxon	Yunnan		Sichuan	References
		E	SE		
*Zosterophyllopsida*	*Demersatheca contigua*		*×*	* *	Li et Cai, 1977; Li et Edwards, 1996
	*Discalis longistipa*		×		Hao, 1989
	*Distichophytum* sp		×		Hao et Xue, 2013
	*Guangnania cuneata*	×	×		Wang et Hao, 2002
	*Guangnania* sp nov.			×	this paper
	*Gumuia zyzzata*		×		Hao, 1989
	*Hicklingia cf. *edwardii*			×	Geng, 1992b
	*Oricilla unilateralis*			×	Geng, 1992b
	*Ramoferis amalia*		×		Hao et Xue, 2011
	*Yunia dichotoma*		×		Hao et Beck, 1991b
	*Yunia guangnania*		×		Hao et Xue, 2013
	*Wenshania zhichangensis*		×		Zhu et Kenrick, 1999
	*Xitunia spinitheca* (L)	×			Xue, 2009
	*Zosterophyllum australianum*	×	×		Li et Cai, 1977; Hao, 1992; Hao et Wang, 2000
	*Z*. *bifurcatum*	×			Li et Cai, 1977
	*Z. *longhuashanense*	×			Li et Cai, 1977
	*Z*. *minifertillum*		×		Hao et Xue, 2013
	*Z*. *minorstachyum* (L)	×			Xue, 2009
	*Z. *myretonianum*	×		×	Li et Cai, 1977; Geng, 1992b
	*Z*. *qujingense*	×			Hao et al., 2007
	*Z*. *ramosum*		×		Hao et Wang, 2000
	*Z*. *shengfengense* (L)	×			Hao et al., 2010
	*Z. *sichuanensis*			×	Geng, 1992b
	*Z*. *spathulatum*	×			Li et Cai, 1977
	*Z*. *tenerum*		×		Hao et Xue, 2013
	*Z*. *xishanense*	×			Hao et al., 2007
	*Z. *yunnanicum*	×			Hsue, 1966; Cai et Schweitzer, 1983, Hao, 1985
Rhyniopsida	? *Hsua deflexa*	×			Wang, Hao et Wang, 2003a,b
	? *Hsua robusta*	×			Li, 1982, 1992
	? *Huia gracilis*	×			Wang et Hao, 2001
	? *Huia recurvata*		×		Geng, 1985
	new taxon			×	this paper
Trimerophytopsida	*Psilophyton primitivum*		×		Hao et Gensel, 1998
	*Pauthecophyton gracile*		×		Xue et al., 2012
	*Yunia dichotoma*		×		Hao et Beck, 1991b
	*Yunia guangnania*		×		Hao et Xue, 2013
Lycopsida	*Baragwanathia* sp.		×		Hao et Gensel, 1998
	*Drepanophycus spinosus			×	Geng, 1992b
	*D*. *qujingensis*	×			Li et Edwards, 1995
	**?** *D*. *spinaeformis*			×	Geng, 1992b
	*Halleophyton zhichangense*		×		Li et Edwards, 1997
	**?** *Hueberia zhichangensis*		×		Yang et al., 2009
	*Leclercqia complexa			×	Geng, 1992b; Xu et Wang, 2009
	*Zhenglia radiate*		×		Hao et al., 2006
Primitive sphenopsids	*Estinnophyton yunnanense*		×		Hao et al., 2004
	**?** *Cervicornus wenshanensis*		×		Li et Hueber, 2000
Barinophytopsida	*Dibracophyton acrovatum*		×		Hao et al., 2012
Incertae sedis	*Adoketophyton subverticillatum*		×		Li et Cai, 1977; Li et Edwards, 1992
	*Adoketophyton parvulum*		×		Zhu et al., 2011
	*Amplectosporangium jiangyouense*			×	Geng, 1992a
	*Bracteophyton variatum*	×			Wang et Hao, 2004
	*Catenalis digitate*		×		Hao et Beck, 1991a
	*Celatheca beckii*		×		Hao et Gensel, 1995
	*Eophyllophyton bellum*		×		Hao, 1988
	*Hedeia sinica*	×	×		Hao et Gensel, 1998
	*Stachyophyton yunnanense*	×			Geng, 1983
	*Polythecophyton demissum*		×		Hao et al., 2001

The latter might apply when differences in ocean currents around a small continent straddling the equator are taken into account ([46], work in progress in Cardiff and Beijing). To add to the enigma, similarities in the spore assemblages between the Sichuan Longmenshan Mountain region and Yunnan and Guangxi provinces which allow correlation would seem to indicate a uniform vegetation and do not reflect the differences seen in the megafossil record although more needs to be known about in situ spores in the endemic taxa (see Stratigraphy section and Supporting Information (SI)

## Conclusions

1. To date, only two taxa in the very diverse Lower Devonian assemblages from northern Sichuan have been investigated in detail. Both are new to science: *Yanmenia longa* is of uncertain affinity, but its leafy nature hints at a lycophyte, while *Guangnania minor* is a new species of the endemic Chinese genus, first recorded from Yunnan Province.
2. A critical preliminary examination of the plants originally described by Geng (Table 1 here) indicates that the majority of the remaining genera described as cosmopolitan has unsafe identifications and thus require re-examination.
3. Comparisons with other assemblages on the South China plate indicate high degrees of endemism in Sichuan (Table 4).
4. Further fieldwork is required to find new localities for megafossils as well as lithologies suitable for preservation of palynomorphs. The latter would allow better correlation and age determinations, although even more valuable would be the discovery of animal fossils to permit dating independent of the plants themselves.
5. Integrated studies involving geologists and palaeontologists are required to develop the Lower Devonian physical and biological history of a fascinating region on the South China plate
